# A comparison of the tablet-based and paper-based versions of the Aberdeen varicose vein questionnaire for quality-of-life assessment in patients with chronic venous disease

**DOI:** 10.1590/1677-5449.202301092

**Published:** 2024-11-22

**Authors:** Daiane Taís Schlindwein Albernaz, Luiz Fernando Albernaz, Fabricio Santiago, Fernanda Rita Zignani, Luís Gustavo Trindade Barroso, Alexandre Reis e Silva, Yung-Wei Chi

**Affiliations:** 1 Clínica Albernaz, Novo Hamburgo, RS, Brasil.; 2 Universidade Federal de Goiás – UFG, Goiânia, GO, Brasil.; 3 Clínica Fleboestética, Feira de Santana, BA, Brasil.; 4 Clínica Reis, Santos, SP, Brasil.; 5 University of California Davis Medical Center, Sacramento, CA, USA.

**Keywords:** venous disease, varicose veins, quality of life questionnaires, Aberdeen varicose vein questionnaire, digital app, doença venosa, veias varicosas, questionários sobre qualidade de vida, questionário de veias varicosas de Aberdeen, aplicativo digital

## Abstract

**Background:**

The Aberdeen Varicose Vein Questionnaire (AVVQ) is the most widely-used questionnaire to assess chronic venous disease. Because the first item requires patients to agree to draw their veins, its paper form has been called into question leading to the development of a tablet version that has simplified its application. However, the literature still lacks a comparison of these tools.

**Objectives:**

To compare agreement between scores, questionnaire completion time, and user-friendliness between paper-based and tablet-based versions of the AVVQ.

**Methods:**

In a prospective, multicenter trial, consecutive patients were asked to complete paper-based and tablet-based versions of the AVVQ. Scores, questionnaire completion time, data entry time, and degree of user difficulty were compared.

**Results:**

Data were collected from 88 patients, 22.7% had completed primary school and 43.2% had higher education. Most patients (88.6%) reported that the tablet version was easy to use. Median time to complete the questionnaire and compute scores was 4 minutes for the tablet version and 9.5 minutes for the paper version (p<0.001). Mean AVVQ scores obtained by patients did not differ significantly between the two groups (p=0.431).

**Conclusions:**

In this study, paper and tablet versions of the AVVQ yielded similar scores, with the tablet version saving time when considering the entire process needed to apply the questionnaire and compute data.

## Resumo

**Contexto:**

O Questionário de Veias Varicosas de Aberdeen (AVVQ) é o questionário mais amplamente utilizado para avaliar doença venosa crônica. Como o primeiro item exige que os pacientes concordem em desenhar suas veias, sua versão em papel foi questionada, levando ao desenvolvimento de uma versão em *tablet* que simplificou sua aplicação. No entanto, a literatura ainda carece de uma comparação dessas ferramentas.

**Objetivos:**

Comparar a concordância entre as pontuações, o tempo de preenchimento do questionário e a facilidade de uso entre as versões em papel e em *tablet* do Questionário de Veias Varicosas de Aberdeen (AVVQ).

**Métodos:**

Em um ensaio multicêntrico prospectivo, foi solicitado que pacientes consecutivos completassem versões em papel e em *tablet* do AVVQ. As pontuações, os tempos de preenchimento, o tempo para inserção de dados e o grau de dificuldade do usuário foram comparados.

**Resultados:**

Os dados de 88 pacientes foram coletados, dos quais 22,7% tinham ensino fundamental completo e 43,2% tinham ensino superior completo. A maioria dos pacientes (88,6%) relatou facilidade de uso da versão em *tablet*. O tempo mediano para completar o questionário e computar as pontuações foi de 4 minutos para a versão em *tablet* e de 9,5 minutos para a versão em papel (p < 0,001). A pontuação média do AVVQ obtida pelos pacientes não apresentou diferença significativa entre os dois grupos (p = 0,431).

**Conclusões:**

Neste estudo, as versões em papel e em *tablet* do AVVQ obtiveram pontuações semelhantes, sendo que a versão em *tablet* apresentou economia de tempo ao considerar o processo completo necessário para aplicar e computar os dados do questionário.

## INTRODUCTION

Chronic venous disease (CVD) is among the most prevalent disorders worldwide.^1^ In the United States, it has been reported that approximately 23% of adults have varicose veins, with 6% experiencing more advanced disease (which includes skin changes and healed or active venous ulcers).^2,^ High CVD prevalence rates have been reported in the literature,^3^ such as 61.3% (C1-C6) in Belgium and Luxembourg,^4^ 69% in Russia,^5^ and over 80% worldwide considering all clinical, etiologic, anatomic, and pathophysiologic (CEAP) classes (C0-C6).^6^ Regardless of clinical class, CVD has been shown to have a negative impact on patients’ daily lives, whether due to symptoms (such as pain and edema) or because of aesthetic concerns.^7^ Several treatment options are available, and most of them seek to improve patient conditions and quality of life.^2^

The use of quality of life questionnaires, both generic and disease-specific, is well established in the literature and supported by guidelines, especially for outcome assessment.^2^ Many studies have shown that patients with varicose veins have worse health-related quality of life than the general population, similar to patients with chronic pulmonary disease, angina, back pain, and arthritis.^8^ Measuring quality of life has gained increasing relevance both as a research outcome and for evaluation of service quality and provider performance, in both public and private healthcare networks.^9^ However, despite the contribution of such tools, they have yet to be incorporated into routine private practice. This may be because of the wide range of questionnaires available, which hinders decision making, as well as the time and effort needed to complete questionnaires and to enter and compute data.

Among disease-specific questionnaires for assessment of CVD, the Aberdeen Varicose Vein Questionnaire (AVVQ) has been evaluated in many different studies,^7^ translated and cross-culturally adapted for use in several countries, and shown to be sensitive for assessing functional outcomes after CVD treatment.^10^

The AVVQ is considered one of the most widely-used CVD questionnaires in the literature.^11^ The first question of the AVVQ assesses the extent of disease, using a schematic illustration on which the patient draws the regions affected by varicosities. The feasibility of this item on the paper form of the questionnaire has been called into question, because it requires patients to agree to draw their veins. A tablet version of the AVVQ has simplified its application and is presented as a solution to this problem. However, the literature still lacks a comparison of these tools, which is the purpose of our research.

The purpose of the present study was to compare agreement between scores, questionnaire completion time, and user-friendliness of the paper-based and tablet-based versions of the AVVQ.

## METHODS

In this prospective, multicenter study conducted at four phlebology clinics located in different cities in Brazil, consecutive patients with CVD were invited to complete the paper-based and tablet-based (mobile app) versions of the AVVQ,^12^ before and after treatment, from January 2016 to December 2017. The QOL ABERDEEN BRA-PRO app (IOS 1.0, version 2016) was developed for use on the tablet device.

As eligibility criteria, all consecutive patients with varicose veins who attended for a scheduled appointment at the following participating clinics were invited to take part: Clínica Albernaz, Novo Hamburgo, RS, Brazil; Universidade Federal de Goiás (UFG), Goiânia, GO, Brazil; Clínica Fleboestética, Feira de Santana, BA, Brazil; Clínica Reis, Santos, SP, Brazil. As such, the applicability of the study to any individual applying for care at a phlebology clinic could be observed.

The inclusion criteria were varicose vein disease regardless of CEAP classification, provision of informed consent, age ≥ 18 years and ≤ 75 years, and ability to read and understand Brazilian Portuguese. All members of the sample selected met the inclusion criteria. The same individuals participated in both groups, completing both questionnaires (paper and tablet) in random order.

Sample size was calculated to detect a 35% difference in standard deviation between the mean scores obtained using the tablet-based or paper-based questionnaires and between the four participating centers, considering an alpha of 0.05 and 90% power. Based on these parameters, 54 patients would be required. Providing for a 10% rate of loss due to missing data, the sample size was defined as 60 patients. Written informed consent was obtained from all individual participants prior to inclusion in the study and the study was approved by the appropriate ethics committee (HU-NH).

### Administration of questionnaires

At each location, all consecutive patients with varicose veins who attended for a scheduled appointment were invited to participate. Patients who met the inclusion criteria completed the paper-based and tablet-based versions of the AVVQ, alternately (one patient completed the paper-based version first, then the tablet-based version; the following patient completed the tablet version first, then the paper version; and so on).

All questionnaires were self-administered. Patients who needed assistance were aided by a clinic receptionist. Assistance was defined as “basic” when limited to verbal instructions or explanations about how to use the tablet device, or “technical” when actual intervention by the receptionist was needed. While completing the questionnaire, patients were assisted by the receptionist, if necessary. Verbal assistance was limited to verbal instructions (explanation on how to use the tablet or how to proceed with the paper questionnaire), while Handling assistance required physical intervention (demonstration of how to manage the tablet or how to manage the paper questionnaire) _(Table 1). The receptionist was restricted to simply providing guidance with handling of the tablet or paper form in case of doubts and was strictly warned that under no circumstances could she participate in the process of answering the questionnaire. If, even after receiving the technical assistance described above, a patient was unable to complete the form on their own, that patient would be considered excluded from the study and notified as such. When a patient brought a companion or chaperone to the appointment, any assistance provided by the chaperone was not considered for analysis. Clinimetric data and ease of administration were assessed on a three-point scale (easy, medium, difficult) by the investigators._

**Table 1:** Table comparing difficulties with completing the questionnaires.

	**Paper questionnaire**	**Tablet**	
Completed successfully	88	88	
Basic assistance	13 (14.8%)	14 (15.9%)	>0.999
Handling assistance	9 (10.2%)	10 (11.4%)	>0.999
Unable to complete by themselves	0	0	

Fisher’s Exact Test.

Data obtained with paper questionnaires were entered into a Microsoft Excel spreadsheet by each investigator, and the time taken to complete data entry was noted. Data obtained with the tablet questionnaires were directly exported to an Excel spreadsheet. The databases from all participating centers were combined and analyzed at the same clinic.

### Statistical analysis

Once the Excel database had been compiled, statistical analyses were performed in PASW Statistics for Windows, version 18.0 (SPSS Inc., Chicago, IL). Categorical variables were expressed as absolute and relative frequencies. Quantitative variables were described as means and standard deviations if symmetrically distributed, or as medians and interquartile ranges otherwise. Scores obtained with the paper and tablet versions of the AVVQ were compared using Student’s *t*-test for paired samples or the Wilcoxon test as appropriate. Pearson’s correlation coefficients were calculated to describe whether the paper and tablet versions were correlated. The Bland-Altman technique was employed to assess agreement between versions and between evaluators. Kappa coefficients were calculated to assess agreement between patients and investigators regarding ease of use. A 5% significance level was considered for all analyses.

## RESULTS

**Figure 1:**
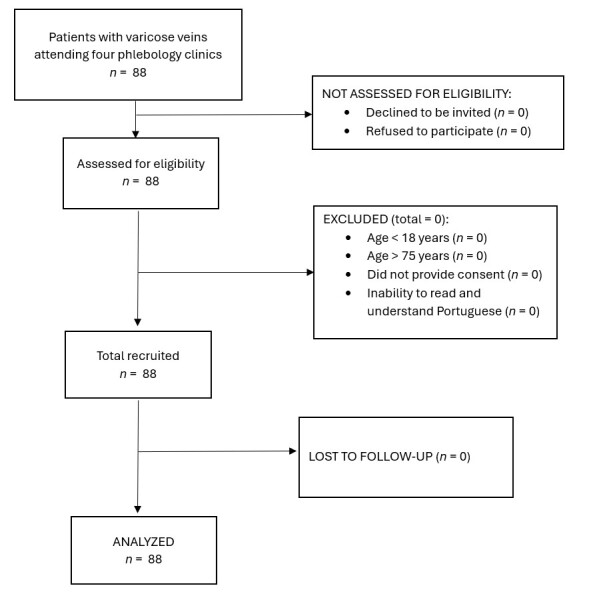
Flow diagram of participant selection.

The flow diagram of participant selection is shown in Figure 1. AVVQ data were obtained from 88 patients, with a mean (SD) age of 46.4 (14.7) years. Regarding educational attainment, 20 patients (22.7%) had completed primary school, 30 (34.1%) had completed secondary school, and 38 (43.2%) had a higher education.

**Figure 2:**
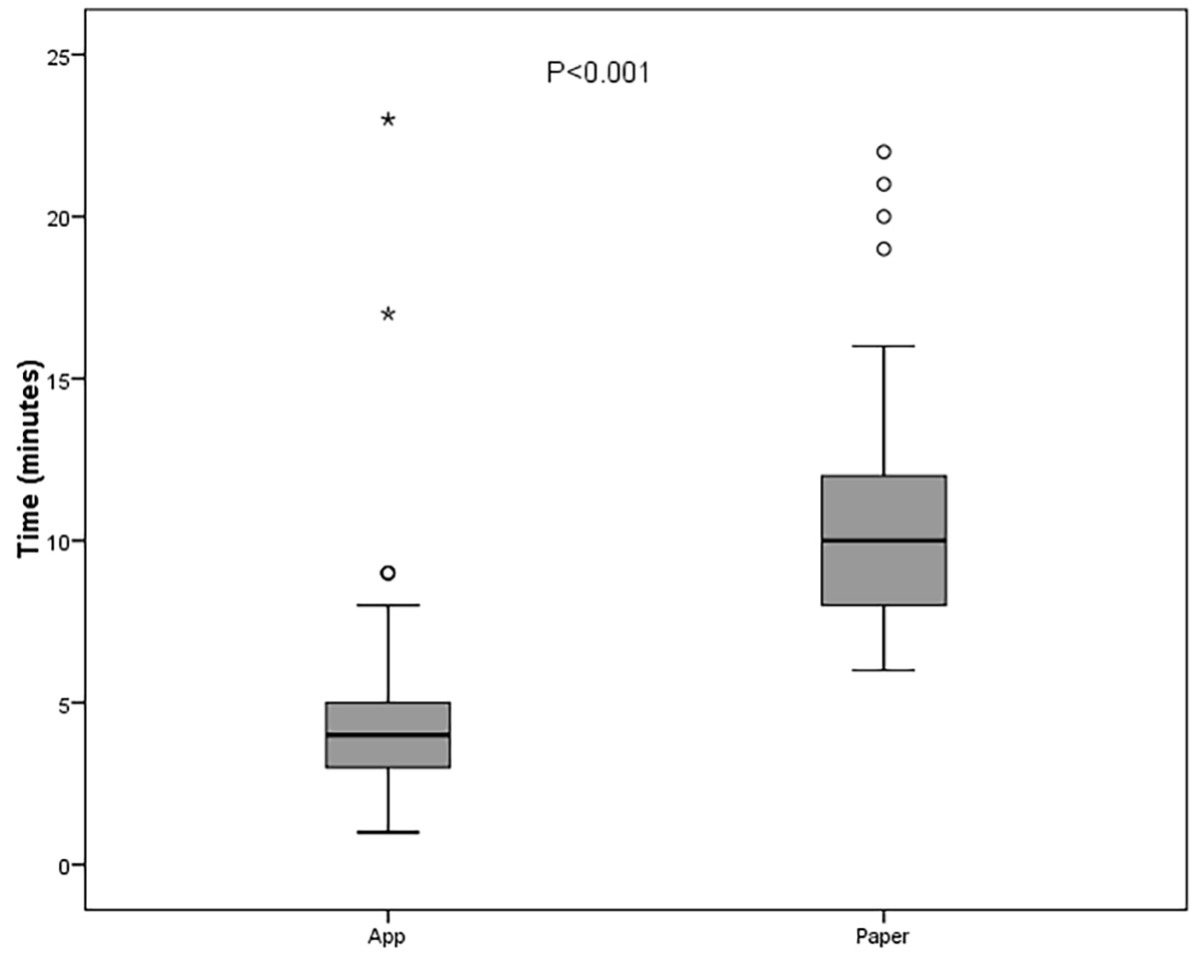
Boxplot comparing time taken to complete questionnaire in tablet and paper versions. *Outliers.

Concerning data entry, the investigators took approximately 2 minutes to transcribe responses from each paper-based questionnaire and 3 minutes to calculate the score for the first question; these times were added to the time taken by each patient to complete the paper-based version of the questionnaire. The median (IQR) time taken to complete the questionnaire and compute scores was 4 (3–5) minutes for the tablet app version and 9.5 (8–12) minutes for the paper-based version (Table 2). Completion time was significantly shorter for the tablet-based version (p<0.001, Wilcoxon test) (Figure 2).

**Table 2:** Time taken to complete questionnaire and compute data for tablet and paper versions.

**Time**	**Tablet app**	**Paper version + transcription**
Mean	4 min 36 s	10 min 7 s
Median	4 min	9 min 30 s
Standard deviation	3 min	3 min 20 s
Minimum	1 min	6 min
Maximum	23 min	22 min
25th percentile	3 min	8 min
75th percentile	5 min	12 min

Overall, 19 patients (21.6%) needed basic assistance: 13 with the paper-based version and 14 with the tablet-based version. Nineteen patients also needed Handling assistance: 9 (47.4%) with the paper version and 10 with the tablet version (52.6%); there was no statistically significant difference between versions. In this sample, all patients were able to complete the questionnaire by themselves. No patients were excluded due to inability to complete the questionnaires.

**Figure 3:**
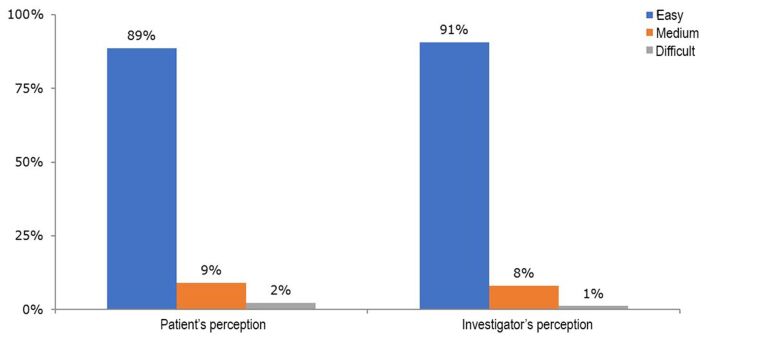
Perceived ease of use of the Aberdeen Varicose Vein Questionnaire according to patients and investigators.

Regarding patients’ impressions of how easy it was to complete the tablet version of the AVVQ, 78 patients (88.6%) classified it as easy, 8 (9.1%) as moderately difficult, and 2 (2.3%) as difficult. In the investigators’ opinion, however, the questionnaire was easy to answer for 80 patients (90.7%), moderately difficult for 7 patients (8.1%) and difficult for only 1 patient (1.2%) (Figure 3). Investigators’ and patients’ opinions of ease of use agreed in 72 cases (83.7%), for a kappa coefficient of 0.15 (p=0.121), denoting a lack of agreement.

There were moderate, inverse, statistically significant correlations between educational level and time to complete both the paper-based and tablet-based versions of the questionnaire. Spearman’s correlation coefficients were -0.43 (p<0.001) for the paper version and -0.42 (p<0.001) for the tablet version, with participants with higher educational level taking less time to complete the questionnaires. There was also a statistically significant correlation between better educational attainment and perceived ease of use by patients (r_s_: -0.35, p=0.001).

The mean (SD) AVVQ score obtained by patients was 17.2 (10.3) for the tablet-based version and 17.7 (10.7) for the paper-based version, with no significant difference between them (p=0.431). The 95% agreement interval for the comparison between the tablet-based and paper-based versions ranged from -10.9 to 10.0 (mean difference, -0.45).

**Figure 4:**
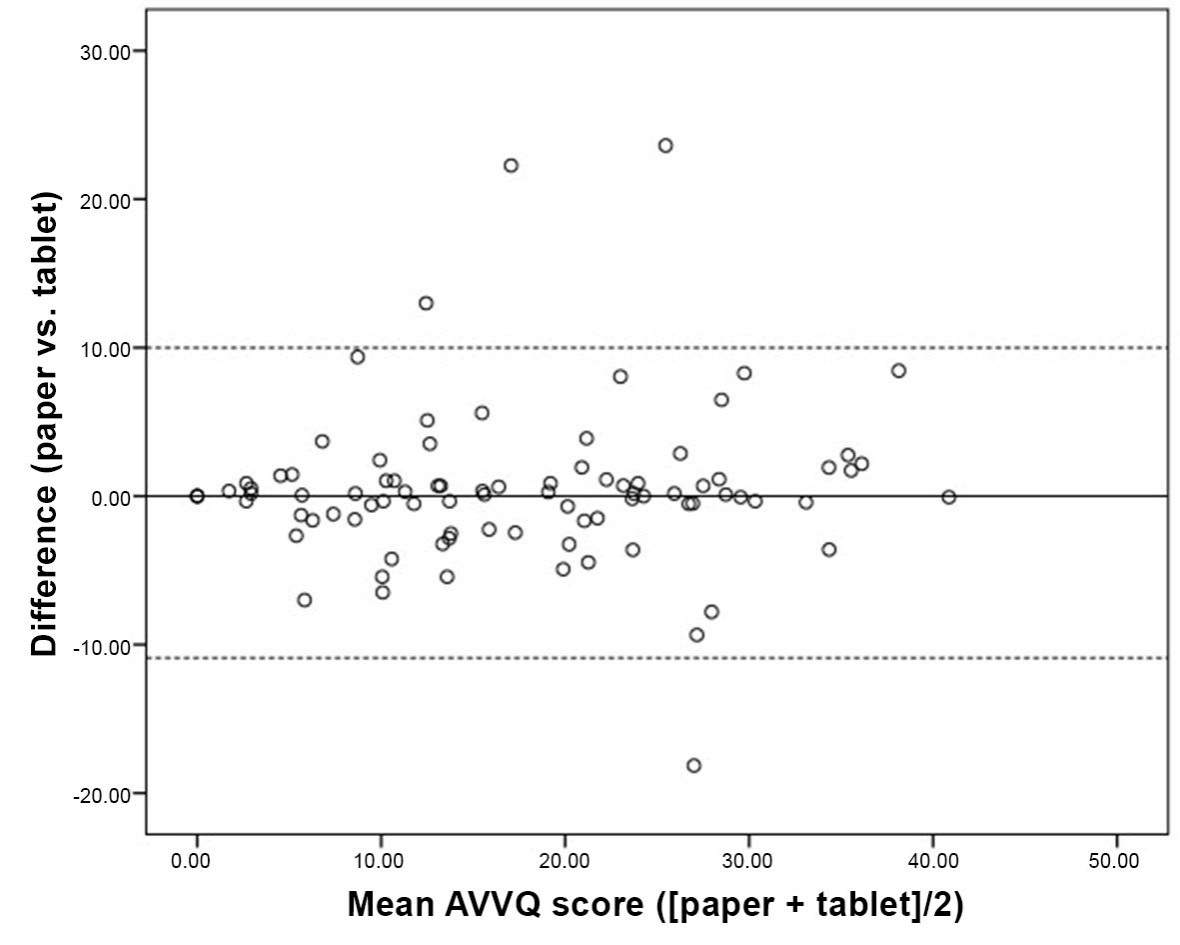
Bland-Altman plot of 95% agreement interval for Aberdeen Varicose Vein Questionnaire scores.

When comparing question 1 of the AVVQ alone, the mean (SD) score was 3.1 (2.3) for the tablet-based version and 3.5 (2.3) for the paper-based version. Although this difference was statistically significant (p=0.011), it was small and does not appear to be clinically relevant. The Bland-Altman plot (Figure 4) illustrates this finding.

## DISCUSSION

As the range of treatments and technologies expands, so does the need to compare outcomes. Surrogate outcomes should be analyzed with care. One limitation of clinical outcome measures is that they do not directly capture the burden of CVD or the patient’s view of its impact on quality of life. Clinically important outcomes are used to measure treatment outcomes, such as improvements in the patient’s function, symptoms, or quality of life and survival.^13^ In this scenario, patient-reported outcomes (PROs) are an essential component of assessment,^14^ and use of disease-specific quality-of-life tools provide a more significant correlate of a patient’s functional status than use of objective anatomic or hemodynamic outcome measures.^10^

Many generic and disease-specific questionnaires for quality-of-life assessment are available in the literature.^2^ However, two issues warrant special attention.

First, the choice of which questionnaire to use should be based on the investigator’s needs. Some questionnaires have been developed for specific conditions, such as ulcers and deep vein thrombosis,^15^ while others encompass a broader spectrum of diseases.^16^

Over the past decade, there has been increasing recognition amongst phlebologists of disease-specific quality-of-life tools.^10^ Alongside the Chronic Venous Insufficiency Questionnaire (CIVIQ) and the Venous Insufficiency Epidemiologic and Economic Study-Quality of Life/Symptom (VEINES-QOL/Sym) instrument, the AVVQ is considered one of the most widely used CVD questionnaires in the literature.^11^ Potential applications for its use range from an impact on health services^17,18^ to cosmetic evaluation in private phlebology practice.^19^ The choice of questionnaire will depend on the purpose to be achieved, considering not only the items but also how the score is calculated. The three questionnaires mentioned above have differences that may play a decisive role in this choice. The VEINES questionnaire uses mean Z scores, which consider the respondent’s performance in relation to his or her group. For this reason, this questionnaire has been widely employed for patient follow-up (e.g., after a thrombotic event). The CIVIQ score and the AVVQ can evaluate individuals with the most varied forms of varicose disease. However, the CIVIQ questionnaire does not include any ulcer-related items. Despite this minor difference, there is a linear correlation between their results.^16^ A recent report positively evaluated the performance of the AVVQ in C1 patients, regardless of item 1 score.^19^

The first question on the AVVQ assesses the extent of disease using a schematic illustration on which the patient draws the regions affected by varicosities. The feasibility of this item on the paper form of the questionnaire has been called into question because it requires patients to agree to draw their veins. Furthermore, it increases the work and time required for data entry, because the patient’s drawing must be converted to a grid on which points will be scored. As a practical solution, the AVVQ score can be calculated even if the first item is left blank. This is presumed to be a disadvantage, but may sometimes be the opposite; when asked to draw their varicose veins, patients are forced to graphically represent their body self-image, which may lead to overestimation or underestimation of disease severity according to the degree of importance the patient ascribes to the disease. Distorted perceptions can change the overall quality of life score.^20^ The graphical representation drawn by the patient from a mental image of his or her varicose veins in the first item confers a psychological dimension on assessment of the extent of varicose disease. Regardless of whether severity is over or underestimated, this will still serve to evaluate outcomes, especially in patients with low CEAP scores. Since the AVVQ assesses PROs, it appears to the authors that this sensitivity of its quality-of-life scores to self-image may differentiate the instrument from photographs or physical examination.^21^

Inclusion of this item in the tablet-based version of the questionnaire required some adaptation. In the app, the schematic drawing used in item 1 is embedded with the grid used to calculate the item score in the paper version of the questionnaire. When the drawing is tapped, the corresponding grid box is highlighted in red and the corresponding region is added instantly to the total score. The ease of completion of this item in the app version may have fully addressed the aforementioned issues of the paper version. In our study, no patients refused to complete item 1 in the paper version of the questionnaire, as reported elsewhere in the literature.^9,^ This fact may be due to the small sample size. More significantly, however, item 1 was completed in all digital questionnaires, and was considered “easy” by 88% of patients. The app version of the questionnaire appears to have solved the issue and restored the importance of measuring the extent of disease in the AVVQ. Lattimer et al.^22^ evaluated the performance of the AVVQ and the venous clinical severity score (VCSS) in the treatment of CVD, observing how patients improve by evaluating the change in each individual question. The largest average variation was found in item 1, where the schematic drawing represented patients’ perceptions of how the disease was being modified by treatment. In this study, the authors advised against omitting the item, a decision which could run contrary to the principles of the AVVQ as a PRO measuring tool.^22^

Despite their importance in outcome assessment and reporting, PRO questionnaires are still essentially restricted to the realm of scientific research or may be used as a guide for patient referral,^23^ with negligible use in private practice or even by specialists.^19,24^ There are occasional reports of their use as population-wide epidemiological measures^25^ and for population monitoring in healthcare systems.^17,26,27^

Second, the mode of data capture. Some reports have considered paper-based instruments as a complicating factor due to the time taken to fill out these forms and subsequently transcribe the collected data. Recently, attempts have been made to allocate efforts toward digitization of paper-based questionnaires,^18^ which may greatly expand their potential utilization by saving time and improving convenience. In the present study, we found statistically significant differences in questionnaire completion and transcription/data entry time between the paper and tablet-based versions of the AVVQ. The median time for tablet data collection (4 minutes) allows us to conclude that the time spent on the questionnaire and on any interventions were not limiting factors for the practical purpose of the study, and that the tablet-based questionnaire was completed faster than the paper-based version, although the difference was small (4 vs. 5 minutes). However, the difference was very significant when the time spent transcribing the paper form to an Excel spreadsheet was included, especially because the first item involves a complex scoring procedure; the median time required to complete the paper version when this step was considered was 9 minutes and 30 seconds per item (Figure 2). Higher educational attainment was associated with shorter time to questionnaire completion for both the paper and mobile app versions.

With regard to difficulties with use of tablets and answering the questionnaire, in this study, patients were invited to complete questionnaires while in the waiting rooms of their respective clinics. Physicians were intentionally not present at the time of questionnaire completion. Any questions were addressed to the clinic receptionist and were considered assistance-seeking behavior. Simple verbal guidance on questionnaire completion was defined as “Verbal assistance”. When intervention from an assistant to demonstrate how to manage the tablet or how to manage the paper questionnaire was deemed imperative for the patient to continue, this was defined as “Handling assistance”. When the patient brought a companion or chaperone to the appointment, any assistance provided by the chaperone was not considered for analysis.

Overall, 19% of patients needed assistance as defined above, half in each group. This stands in contrast to the assessment of ease of use, where 89% of patients found the questionnaire “easy” to answer. Very few patients actually required intervention (Handling assistance) and this was not a factor limiting questionnaire completion, as no patients were excluded due to inability to complete the questionnaire. Educational level was directly associated with time to questionnaire completion and correlated inversely with the perceived degree of difficulty; therefore, considering that 22% of the participants only had primary education, the overall degree of difficulty was indeed low.

The mean (SD) AVVQ score obtained by patients was 17.2 (10.3) with the mobile app versus 17.7 (10.7) with the paper version; there was no statistically significant difference between them (p=0.431). The 95% agreement interval between the two versions ranged from -10.9 to 10.0, with a mean difference of -0.45. These data indicate that use of a digital interface does not impair the information reported by the patient and may validate the use of a tablet device as an option as reliable as the paper version.

## CONCLUSION

In this study, both paper and tablet versions of the AVVQ yielded similar scores, with the tablet version saving time when considering the entire process needed to apply the questionnaire and compute data. These findings suggest that practical, time-saving versions of PRO measurement instruments may become part of routine clinical care both in public healthcare and in private practice, contributing numerical results to the constant search for evidence.
